# Evaluation of plasma brain-derived neurotrophic factor levels and self-perceived cognitive impairment post-chemotherapy: a longitudinal study

**DOI:** 10.1186/s12885-017-3861-9

**Published:** 2017-12-19

**Authors:** Terence Ng, Ying Yun Lee, Jung-woo Chae, Angie Hui Ling Yeo, Maung Shwe, Yan Xiang Gan, Raymond C. H. Ng, Pat Pak Yan Chu, Chiea Chuen Khor, Han Kiat Ho, Alexandre Chan

**Affiliations:** 1 Department of Pharmacy, Faculty of Science, National University of Singapore, Block S4A, 18 Science Drive 4, Level 3, Singapore, 117543 Singapore; 2 Department of Pharmacy, National Cancer Centre Singapore, Singapore, Singapore; 3 Duke-NUS Medical School Singapore, Singapore, Singapore; 4 Division of Medical Oncology, National Cancer Centre Singapore, Singapore, Singapore; 5 Singapore Cord Blood Bank, K.K. Women’s and Children’s Hospital, Singapore, Singapore; 6 Genome Institute of Singapore, Singapore, Singapore

**Keywords:** BDNF, Breast cancer, Cognition, Genetics, rs6265

## Abstract

**Background:**

Preliminary evidence suggests that changes in plasma brain-derived neurotrophic factor (BDNF) levels may contribute to the occurrence of chemotherapy-associated cognitive impairment (CACI), and a previous study suggested that carriers of the BDNF Met homozygous genotype are protected from CACI.

**Methods:**

This multicenter, prospective cohort study involved chemotherapy-receiving early-stage breast cancer (ESBC) patients. Self-perceived cognitive function was longitudinally assessed using the validated FACT-Cog (ver. 3) across three time points: Prior to chemotherapy (T1), during chemotherapy (T2), and at the end of chemotherapy (T3). Plasma BDNF levels were quantified using enzyme-linked immunosorbent assay. Genotyping was performed using Sanger Sequencing.

**Results:**

A total of 51 chemotherapy-receiving ESBC patients (mean age: 52.6 ± 9.5 years) were recruited, and 11 patients (21.6%) reported subjective cognitive impairment post-chemotherapy. Overall, there was a reduction in median plasma BDNF levels over time (T1: 5423.0 pg/ml; T2: 5313.6 pg/ml; T3: 4050.3 pg/ml; *p* < 0.01). After adjusting for confounding factors, longitudinal analysis revealed that BDNF levels were associated with self-reported concentration deficit (*p* = 0.032). Carriers of Val/Val (*p* = 0.011) and Val/Met (*p* = 0.003) BDNF genotypes demonstrated a significant reduction in plasma BDNF levels over time; however, plasma BDNF levels were similar across all time points among Met homozygous carriers (*p* = 0.107).

**Conclusion:**

There was a statistically significant change in BDNF levels post-chemotherapy in ESBC patients, and plasma BDNF levels were associated with self-perceived concentration deficit in patients receiving chemotherapy.

## Background

Chemotherapy-associated cognitive impairment (CACI) among breast cancer survivors has been widely reported [1]. Often termed as “*chemobrain,*” cognitive changes are subtle, yet notable. Memory, attention, and executive function are particularly susceptible to chemotherapy-induced changes, and these changes may adversely affect a patient’s daily functioning and quality of life.

While numerous mechanisms to explain chemobrain have been postulated, including direct chemotherapy-induced toxicities, immunologic alterations, and neural repair insufficiencies, little is definitively known about its actual causes. Of the proposed mechanisms, it has been suggested that the brain-derived neurotrophic factor (BDNF) may be implicated in CACI. BDNF is a type of neurotrophin extensively distributed in the central nervous system, particularly in the prefrontal cortex and hippocampus [2–4]. Through its action on tropomyosin-related kinase B receptors [5], BDNF plays an essential role in regulating synaptic plasticity, neuronal growth, and survival [3, 4, 6]. In particular, it has been noted for its involvement in neurotransmitter release and long-term potentiation (LTP) [3, 4]. Long-term potentiation is important to memory and learning, and the inhibition of LTP may result in hippocampal-dependent memory impairment [7].

Numerous studies have reported the possible role of BDNF in the pathogenesis of various cognitive disorders, such as Alzheimer’s disease [3, 4, 6, 8, 9]. Low serum BDNF levels have been correlated with Alzheimer’s disease and mild cognitive impairment, and high serum BDNF levels have been associated with better cognition in healthy older adults. Studies have also suggested that plasma BDNF reflects cortical BDNF signaling during learning in healthy adults [10]. Consistent with the increasing evidence for BDNF’s role in cognition, studies have also noted the contribution of BDNF Val66Met polymorphism (rs6265) in cognitive function and various neuropsychiatric disorders [11]. Our research group recently revealed that rs6265 confers a protective effect against CACI in the early-stage breast cancer (ESBC) population [12]. However, the trajectory of plasma BDNF during chemotherapy and its relation with rs6265 remain unknown.

Given the possible implication of BDNF in cognition, it is worthwhile exploring the possible association of peripheral BDNF levels and cognitive function in the chemotherapy-receiving cancer population. Hence, this pilot study was designed to investigate the changes of plasma BDNF levels and self-perceived cognitive impairment in ESBC patients receiving chemotherapy. A secondary objective of this study was to investigate the differences in plasma BDNF levels between the rs6265 genotypes.

## Methods

### Study design and participants

This was a prospective cohort study conducted at the National Cancer Centre Singapore and KK Women’s and Children’s Hospital between November 2014 to December 2015. This study was conducted in accordance to the Declaration of Helsinki and approved by SingHealth Institutional Review Board (CIRB 2014/754/B). Patients were given informed consent before recruitment. The inclusion criteria of this study were: (1) age ≥ 21 years, (2) understood English or Mandarin, (3) diagnosed with ESBC (stage I-IIIa), (4) scheduled for anthracycline- or taxane-based chemotherapy, and (5) had no prior history of chemotherapy and/or radiotherapy. Exclusion criteria include (1) incapable of giving informed consent, (2) symptomatically ill, (3) presence of neuropsychiatric disorders, and (4) presence of neurologic or immune-related conditions.

### Procedures

At the point of recruitment, patients’ demographics and medical information were obtained via electronic medical records and patient interviews. Patients’ self-perceived cognitive function and behavioral symptoms were assessed using subjective assessment tools at three time points in approximately 6-week intervals: baseline (T1), during (T2) and at the end of chemotherapy (T3). The assessments were conducted either in English or Chinese by trained personnel. Each session was approximately 45 min. At each time point, 10 mL of whole blood was collected in a heparinized-tube and immediately centrifuged at 2500 rpm for 10 min. The plasma and buffy coat were stored at −80 °C until analysis.

### Assessment of self-perceived cognitive impairment

The Functional Assessment of Cancer Therapy-Cognitive Function (FACT-Cog) version is a 37-item questionnaire which evaluates patients’ self-perceived cognitive decline within the past 7 days, and this tool has been validated for usage in our population [13]. Six domains of cognition (mental acuity, attention and concentration, memory, verbal fluency, functional interference and multitasking ability) are evaluated on a scale of 0 (“Never” or “Not at all”) to 4 (“Several times a day” or “Very much”). Reverse scores from individual items are tallied to obtain the global FACT-Cog score. A higher score denotes better self-perceived cognitive function. The patients were classified as having self-perceived cognitive impairment if there was a reduction of at least 10.6 points in the global FACT-Cog score at T2 or T3 relative to the baseline value. For each cognitive subdomain, a 15% decrease score reduction at T2 or T3 relative to the baseline value is considered as impairment. This classification has been utilized in other studies that evaluated self-perceived cognitive impairment in cancer patients [12, 14].

### Assessment of cancer-related fatigue

Cancer-related fatigue was assessed using the Brief Fatigue Inventory (BFI) [15]. The BFI measures the severity of fatigue (a known confounder of cognition) at the point of assessment and in the past 24 h, rated in a scale of 0 (“no fatigue”) to 10 (“fatigue as bad as you can imagine”). BFI assesses whether fatigue interfere on daily activities in the past 24 h, on a scale of 0 (“does not interfere”) to 10 (“completely interferes”). Six aspects of interference were assessed: general activity, mood, walking ability, normal work, relations with other people and enjoyment of life.

### Assessment of anxiety

Anxiety was assessed using the Beck Anxiety Inventory (BAI), and this tool has been validated for usage in our population [16]. The BAI measures the severity of anxiety, a known confounder of cognition, in the past month based on 21 items listing common anxiety symptoms, on a scale of 0 (“not at all”) to 3 (“severe”). Scores from individual items were tallied to obtain a global score. A higher score denotes greater anxiety.

### Assessment of depression

Depression was assessed using the Beck Depression Inventory (BDI) [17]. The BDI evaluates 21 symptoms of depression (14 cognitive-affective symptoms, 7 somatic symptoms) on a four-point intensity scale. Scores were tallied to a total score. A higher score reflects greater severity of depression.

### Plasma BDNF quantification

Plasma BDNF levels were analyzed in duplicate using a commercial enzyme-linked immunosorbent assay (ELISA) kit (Biosensis® BEK-2211-2P, Australia) according to the manufacturer’s instructions. The kit detects 100% of the mature BDNF, with less than 7% cross-reactivity with the pro-form of BDNF. Brain-derived neurotrophic factor standards (7.8–500.0 pg/mL) were prepared via serial dilutions, and the plasma samples underwent dilution by a factor of 50. Brain-derived neurotrophic factor was quantified at 450 nm using a plate reader. The standard curve was constructed using a four-parameter logistic model using ElisaAnalysis.com (Leading Technology Group, Australia) software. An intra-assay coefficient of variance (CV) of less than 10% was considered to be acceptable.

### Genotyping

Genomic DNA from the buffy coat was isolated using a QIAamp DNA Blood Mini Kit (Qiagen, Germany). The region containing the BDNF rs6265 polymorphism was amplified by polymerase chain reaction (PCR) using specific and optimized primers. The primers involved were: 5′-GGACTCTGGAGAGCGTGAA-3′ (forward) and 5′-CGTGTACAAGTCTGCGTCCT-3 (reverse). Genotyping of the PCR products was performed by automated Sanger sequencing using a 3730xl DNA Analyzer (Applied Biosystems, United States). Samples were identified only by codes, and genotyping was blindly performed by AITbiotech without knowledge of the clinical outcomes.

### Statistical analysis

All of the statistical analyses were performed using Stata Version 14 (StataCorp, 2015). Descriptive statistics were utilized to summarize the demographic and clinical characteristics of the patients. The Friedman test was utilized to evaluate changes in the plasma BDNF levels over time, and the post-hoc Wilcoxon signed-rank test was used for pair-wise comparisons between individual time points. Deviation of the genotypes from Hardy-Weinberg equilibrium was calculated using the chi-squared test with one degree of freedom.

To investigate the association between plasma BDNF levels and self-perceived cognitive function (overall and each of the six domains) over time, we created a generalized estimating equations (GEE) model. Selection of the appropriate correlation structures in the GEE model was conducted using the quasi-likelihood under the independence model criterion, the structure exhibiting the smallest criterion was considered as the most desirable. Documented confounders of BDNF levels and self-perceived CACI (fatigue, anxiety, depression, age, body mass index (BMI) and BDNF genotypes) were included in the statistical model [12, 18].

Subgroup analyses were performed to determine the change in plasma BDNF levels after classifying for self-perceived cognitive impairment and the BDNF rs6265 genotypes. The Kruskal-Wallis test and Mann-Whitney U test were used to analyze cross-sectional differences in plasma BDNF levels between the genotypes and to analyze differences in plasma BDNF levels between cognitively impaired and non-impaired population. All of the statistical tests were two-sided, and *p* < 0.05 was considered to correspond to statistical significance.

## Results

### Demographics and clinical information

The analysis included 51 ESBC patients with a mean age of 52.6 ± 9.5 years (Table 1). The patients were predominantly Chinese (78.4%), and 44 patients (86.2%) had completed at least secondary school. Thirty-two patients (62.8%) were diagnosed with stage II breast cancer. All of the patients were ambulatory without activity restrictions. Twenty-nine patients (56.9%) received anthracycline-based chemotherapy, and 22 patients (43.1%) received taxane-based chemotherapy. Using the Minimal Clinical Important Difference (MCID) of the FACT-Cog, 11 patients (21.6%) were classified as manifesting self-perceived cognitive impairment.

**Table 1:** Demographics and clinical information of patients (*n* = 51)

Characteristics		*n* (%)
Age (years, mean ± SD)		52.6 ± 9.5
Race	Chinese	40 (78.4)
	Malay	4 (7.8)
	Indian	6 (11.8)
	Other	1 (2.0)
Marital status	Single	8 (15.7)
	Married	40 (78.4)
	Divorced	3 (5.9)
Education level	None	1 (2.0)
	Primary school	6 (11.8)
	Secondary school	22 (43.1)
	Pre-university	7 (13.7)
	Graduate/postgraduate	15 (29.4)
Occupation	Currently working	36 (70.6)
	Currently not working	13 (25.4)
	Retired	1 (2.0)
	Long-term medical leave	1 (2.0)
Cancer diagnosis	Stage I	7 (13.7)
	Stage II	32 (62.8)
	Stage III	12 (23.5)
ECOG Performance status	0	51 (100.0)
Menopausal status	Pre-menopausal	20 (39.2)
	Post-menopausal	31 (60.8)
Chemotherapy regimen	Anthracycline-based	29 (56.9)
	Taxane-based	22 (43.1)
Behavioral symptoms, median (IQR)		
Baseline fatigue (BFI total score, out of 10)		0.7 (0.1,1.8)
Baseline anxiety (BAI total score, out of 63)		0.0 (0.0,0.0)
Baseline depression (BDI total score, out of 63)		3.0 (1.0,8.0)

*Abbreviations*: *SD* standard deviation, *IQR* interquartile range, *ECOG* Eastern Cooperative Oncology Group, *BFI* Brief Fatigue Inventory, *BAI* Beck Anxiety Inventory, *BDI* Beck Depression Inventory

Analysis of the behavioral symptoms revealed a statistical significant increase in median fatigue levels (0.67 at T1 vs. 1.67 at T3, *p* < 0.001). Median anxiety levels, as measured by BAI, increased from 0 at T1 to 6 at T3 (*p* = 0.040), while depression severity, as measured by BDI, increased from a median score of 3 at T1 to 6 at T3 (*p* < 0.001).

### Genotypes and allele frequencies

Fifty patients were successfully genotyped for the BDNF rs6265 polymorphism. The genotype frequency did not deviate from the Hardy-Weinberg Equilibrium (χ^2^ = 0.07, *p* > 0.05). The Val (52.0%) and Met (48.0%) allele frequency was approximately equivalent. (Table 2).

**Table 2:** Genotype and allele frequencies of the BDNF Val66Met polymorphism (*N* = 50)^a^

	Population, n (%)				Pooled Asians, *n* (%)
	Chinese	Malay	Indian^a^	Others^b^	
	(n=40)	(n=4)	(n=5)	(n=1)	(n=50)
Genotype frequency					
GG (Val/Val)	11 (27.5)	1 (25.0)	2 (40.0)	0 (0.0)	14 (28.0)
GA (Val/Met)	18 (45.0)	3 (75.0)	2 (40.0)	1 (100.0)	24 (48.0)
AA (Met/Met)	11 (27.5)	0 (0.0)	1 (20.0)	0 (0.0)	12 (24.0)
Allele frequency					
G (Val) allele	40 (50.0)	5 (62.5)	6 (60.0)	1 (50.0)	52 (52.0)
A (Met) allele	40 (50.0)	3 (37.5)	4 (40.0)	1 (50.0)	48 (48.0)

^a^Genotype data for one patient is not available

^b^Others include 1 Filipino

### Trajectory of plasma BDNF levels over time

**Fig. 1:**
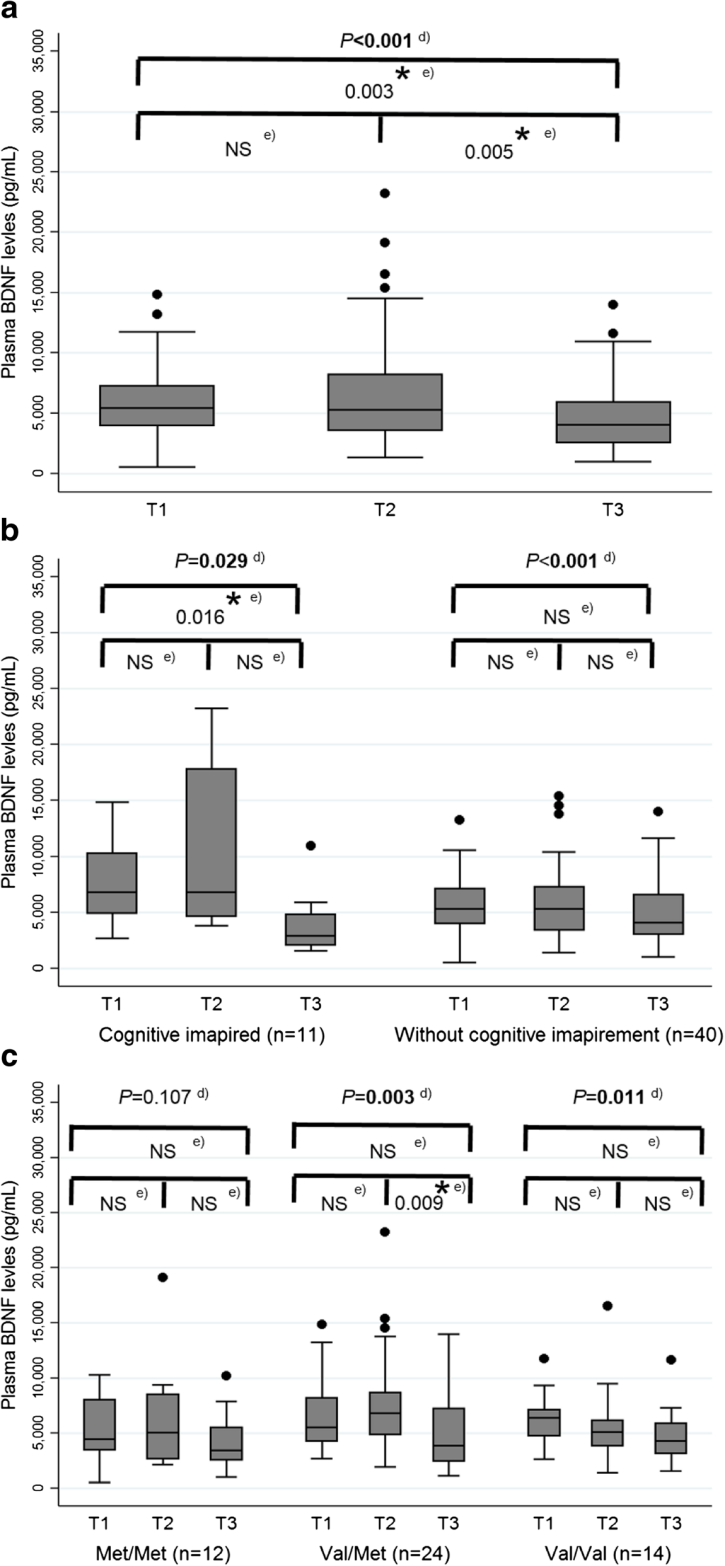
Trajectory of plasma BDNF levels over time (*n* = 51). **a** Classified based on the entire patient pool; **b** classified based on overall cognition status; **c** classified based on BDNF rs6265 genotypes; **d** the Friedman test was used to evaluate changes in the plasma BDNF levels over time, **e** Post-hoc analysis was conducted using the Wilcoxon signed rank test for pair-wise comparisons. ^*^ *P* values are significant if they are less than 0.0167

All of the plasma BDNF levels fell within the BDNF standard curve (7.8–500.0 pg/mL) after dilution. The range of BDNF levels detected with ELISA, after correcting for the dilution factor, was 538.6–23,218.7 pg/mL. The mean intra-assay CV obtained was 4.9%. There was a statistically significant difference in BDNF levels across the three time points (T1: 5423.0 vs. T2: 5313.6 vs. T3: 4050.3 pg/mL; *p* < 0.001), with a decreasing trend over time (Fig. 1a).

Among individuals who were cognitively impaired (*n* = 11), BDNF levels were statistically different over time (T1: 5423.0 vs. T2: 5823.2 vs. T3: 3095.7 pg/mL; *p* = 0.029) (Fig. 1b). A post-hoc comparison of BDNF levels at T3 versus T1 was statistically significant (*p* = 0.016). Similarly, non-impaired individuals (*n* = 40) experienced a significant reduction in BDNF levels across the three-time points (*p* < 0.001).

A comparison of plasma BDNF levels at baseline did not reveal statistically significant difference between self-perceived cognitively impaired (5423.0 pg/mL) and non-impaired individuals (5430.0 pg/mL) (*p* = 0.664). Similarly, plasma BDNF levels were similar between the impaired and non-impaired populations during chemotherapy (5823.2 pg/mL vs. 5313.6 pg/mL; *p* = 0.309) and after chemotherapy (3095.7 vs. 4069.5 pg/mL; *p* = 0.336).

### Associations between plasma BDNF levels and self-perceived CACI

After accounting for known confounders of self-reported cognitive impairment, the GEE model revealed that BDNF levels were found to be associated with self-perceived concentration deficit. (Table 3). BAI was associated with the proportion of overall self-perceived cognitively impaired individuals over time (*p* < 0.001). BAI was also associated with self-perceived mental acuity deficit (*p* = 0.001), self-perceived concentration deficit (*p* = 0.047), self-perceived memory deficit (*p* = 0.007), self-perceived verbal fluency interference (*p* = 0.001), self-perceived functional interference (*p* = 0.006) and self-perceived multi-tasking ability interference (*p* = 0.001), while BFI was associated with self-perceived concentration deficit (*p* < 0.001), self-perceived memory deficit (*p* = 0.009) and self-perceived functional interference (*p* = 0.040).

**Table 3:** Association between proportion of individuals with self-perceived cognitive impairment and plasma BDNF levels (*n* = 51)

Variable	Proportion of individuals with cognitive impairment^a^		FACT-Cog domains^b^											
			Mental Acuity		Concentration		Memory		Verbal		Functional		Multi-tasking	
	Coefficient (SE)	*p* value^c^	Coefficient (SE)	*p* value^c^	Coefficient (SE)	*p* value^c^	Coefficient (SE)	*p* value^c^	Coefficient (SE)	*p* value^c^	Coefficient (SE)	*p* value^c^	Coefficient s(SE)	*p* value^c^
BDNF^d^	0.0001 (0.00007)	0.108	−0.00004 (0.0001)	0.669	0.00005 (0.00002)	**0.032**	−0.00003 (0.00009)	0.732	0.00005 (0.00007)	0.511	−0.00006 (0.0001)	0.617	0.00009 (0.00006)	0.138
BFI	0.339 (0.234)	0.147	0.096 (0.250)	0.700	−0.367 (0.067)	**<0.001**	0.652 (0.249)	**0.009**	0.314 (0.222)	0.157	0.708 (0.345)	**0.040**	0.274 (0.190)	0.149
BAI	0.241 (0.072)	**0.001**	0.275 (0.082)	**0.001**	−0.042 (0.021)	**0.047**	0.201 (0.074)	**0.007**	0.247 (0.072)	**0.001**	0.308 (0.112)	**0.006**	0.196 (0.059)	**0.001**
BDI	−0.005 (0.085)	0.949	−0.010 (0.093)	0.912	−0.063 (0.027)	**0.018**	−0.090 (0.097)	0.355	−0.079 (0.089)	0.370	−0.116 (0.123)	0.343	−0.033 (0.077)	0.667
Age	0.037 (0.044)	0.403	0.010 (0.044)	0.830	0.011 (0.025)	0.669	−0.010 (0.051)	0.848	0.027 (0.045)	0.550	−0.009 (0.065)	0.888	0.037 (0.044)	0.397
BMI	0.141 (0.076)	0.064	0.148 (0.078)	0.056	−0.006 (0.048)	0.907	0.116 (0.082)	0.156	0.097 (0.078)	0.213	0.152 (0.094)	0.105	0.078 (0.077)	0.312
BDNF genotype^e^	−1.191 (0.582)	**0.041**	−0.543 (0.582)	0.351	−0.658 (0.326)	**0.043**	−0.233 (0.698)	0.738	−0.811 (0.591)	0.170	−0.400 (0.887)	0.652	−0.698 (0.554)	0.208
Constant	−8.570 (3.628)	**0.018**	−7.600 (3.825)	**0.047**	16.035 (1.764)	**<0.001**	−6.742 (4.023)	0.094	−6.655 (3.645)	0.068	−8.930 (5.134)	0.082	−6.746 (3.546)	0.057

*Abbreviation*: *SE* standard error, *BFI* Brief Fatigue Inventory, *BAI* Brief Anxiety Inventory, *BDI* Beck Depression Inventory, *BMI* body mass index

^a^Proportion of individuals with cognitive impairment is estimated based on the Minimal Clinically Important Difference of the global FACT-Cog score. Patients with a FACT-Cog score of more than −10.6 points from baseline were classified as having cognitive impairment

^b^Proportion of individuals with impairment at the respective domains is determined by a MCID of 15% decrease in the FACT-Cog subdomain scores from baseline

^c^Bolded p values indicate statistical significance

^d^Plasma BDNF levels were ranged in pg/mL

^e^Reference genotype is Val/Val

The BDNF Met risk allele was found to be associated with both overall self-perceived cognitive impairment (*p* = 0.041) and self-perceived concentration deficit (*p* = 0.043). There were no statistically significant associations between self-perceived cognitive disturbances with age and BMI.

### Change of plasma BDNF levels in relation to BDNF Val66Met polymorphism classification

There was a statistically significant change in plasma BDNF levels over time among the Val/Val (*p* = 0.011) and Val/Met genotype (*p* = 0.003); however, the change was not statistically significant among carriers of the Met/Met genotype (*p* = 0.107) (Fig. 1c).

## Discussion

Given the role of BDNF in mediating synaptic plasticity and neuronal growth in the hippocampus, studies have suggested BDNF’s involvement in cognitive function, particularly in learning and memory [3, 4, 6, 7]. This study sought to investigate the changes of plasma BDNF levels and self-perceived cognitive impairment in ESBC patients receiving chemotherapy. We have observed a statistically significant change in plasma BDNF levels over time post-chemotherapy, and such observation was relevant in both self-perceived cognitive impaired and non-impaired subgroups. To further elucidate the relationship between BDNF levels and self-perceived cognitive impairment, after adjusting for known confounders (including BFI, BAI, BDI, age, BMI and BDNF genotypes) of self-perceived cognitive impairment, our longitudinal model revealed that BDNF levels were associated with self-perceived concentration deficit. However, changes in BDNF levels were not observed among those who were Met homozygous carriers of the BDNF genotype.

Although we have observed an association between BDNF levels and self-perceived concentration deficit, the findings are not consistent with other published studies. Such inconsistencies could be attributed to differences in the study designs, disease populations, and cognitive assessment tools. Previous studies were mainly cross-sectional in nature, and they were conducted in non-cancerous populations. A number of longitudinal studies did not establish any associations between serum BDNF levels and cognitive decline in healthy older adults [19, 20]. However, one study revealed that BDNF levels in the cerebrospinal fluid (CSF) were significantly associated with greater decline in cognitive function, specifically within the memory domain in healthy older adults [21]. This finding suggests that plasma BDNF levels may not be fully representative of the CSF BDNF levels in the brain. Although BDNF is able to cross the blood-brain barrier [22], the degree to which plasma BDNF levels can represent CSF BDNF levels remains unclear [23, 24]. Further investigations have to be conducted to draw a conclusion on the use of plasma BDNF levels as a surrogate marker for CSF BDNF levels.

**Fig. 2:**
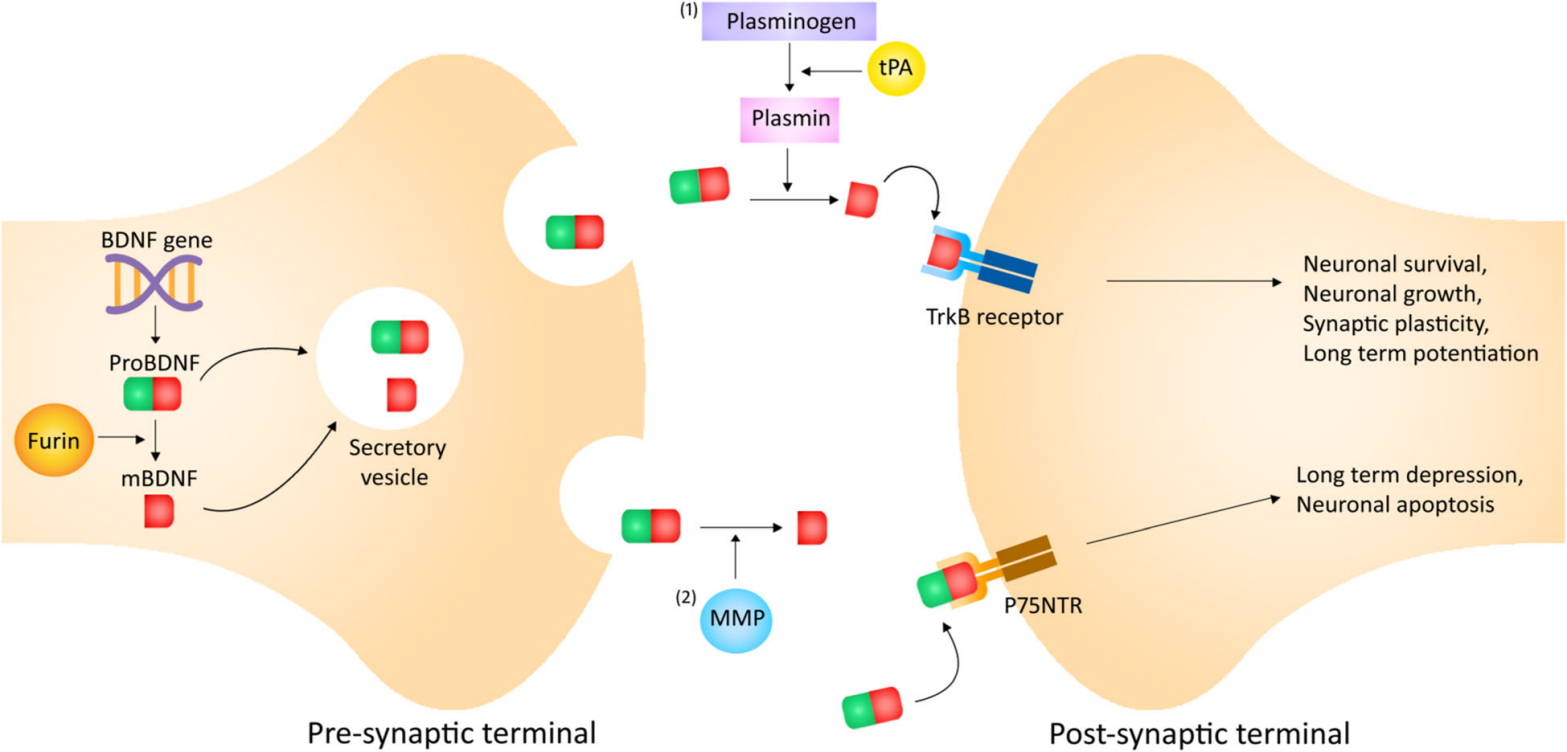
Schematic illustration of the biological processing of BDNF. Abbreviation: BDNF, brain-derived neurotrophic factor; mBDNF, mature brain-derived neurotrophic factor; tPA, tissue plasminogen activator; MMP, matrix metalloproteinases; TrkB, tropomyosin-related kinase B; P75NTR, p75 neurotrophin receptor

Emerging studies have highlighted the contrasting roles of BDNF isoforms in cognition [25–28]. Biologically, BDNF is synthesized as a precursor known as proBDNF prior to cleavage into the mature BDNF (mBDNF) via furin intracellularly or matrix metalloproteinases (MMP) and plasmin extracellularly [26, 27, 29] (Fig. 2). ProBDNF is necessary for promoting the folding of the mature domain and for sorting BDNF into secretory vesicles. While mBDNF regulates neuronal growth and survival, proBDNF preferentially binds to p75 neurotrophin receptor (p75NTR) to activate neuronal apoptotic pathways [27, 30]. In a sample of autistic patients, it was suggested that an imbalance in BDNF isoforms may have been a possible mechanism leading to autism [25]. High plasma BDNF levels may not indicate better cognition if proBDNF predominates over BDNF, which results in greater neuronal apoptosis. Another study revealed that the direction of BDNF regulation could be affected by mechanisms controlling the cleavage of proBDNF [31]. High-frequency stimulation was found to promote the release of proBDNF and tissue plasminogen activator; low-frequency stimulation only resulted in proBDNF release. However, this study did not evaluate the expressions levels of proBDNF. Future studies should evaluate the ratio between mBDNF and proBDNF in the context of CACI.

In this study, the Met risk allele is associated with both overall self-perceived cognitive impairment and self-perceived concentration deficit. This is in agreement with the literature that the BDNF Val66Met polymorphism (rs6265) may contribute to varying cognitive function. One study has suggested that the Met allele confers a protective effect against CACI in patients receiving chemotherapy, specifically in the areas of verbal fluency and multitasking ability [12]. The BDNF Val66Met polymorphism involves a non-synonymous single nucleotide polymorphism (SNP) that results in the substitution of Valine to Methionine at codon66 at the proBDNF region. As a result of this change, there is an impaired sorting of BDNF into secretory vesicles [11]. This impairment might lead to a reduction of proBDNF release, which is the major BDNF isoform present in secretory vesicles. Consequently, carriers of the Met allele would express lower baseline levels of proBDNF compared to the carriers of the Val allele. It is unknown, however, whether reduced level of proBDNF could affect mBDNF levels by other compensatory mechanisms. Further studies are required in order to elucidate the relationship between BDNF polymorphism and proBDNF expression in the CACI setting.

The strengths of this study include its prospective nature and the longitudinal assessment of cognitive function and behavioral symptoms. Since the manifestation of CACI is complex and can be influenced by multiple factors, documented confounders [12, 18] such as anxiety, fatigue and depression (using BAI, BFI and BDI) were also evaluated in this study. However, one major limitation was the relatively small sample size that subjects this study to Type 2 errors. A study with larger sample size is required to confirm this finding. This study also lacks an objective assessment of cognitive function using neuropsychological batteries, as recommended by the International Cognition and Cancer Task Force [32]. A larger sample size, as well as a replication of the findings of this study in an independent cohort at a different site deems important. Hence, future studies should validate the findings of this study.

## Conclusions

In this pilot study, we have observed a statistically significant change in BDNF levels post-chemotherapy. After adjusting for potential confounders, a change in BDNF levels was associated with self-perceived concentration deficit. BDNF levels, however, remain similar over time among carriers of the Met homozygous carriers of the BDNF rs6265 polymorphism. Given the complexity of BDNF and cognitive function, additional studies taking into account proBDNF, MMP, and plasmin should be conducted in order to gain a better understanding of the contribution of the various BDNF isoforms to CACI.

## Abbreviations

BAI: Beck Anxiety Inventory
BDI: Beck Depression Inventory
BDNF: Brain-derived neurotrophic factor
BFI: Brief Fatigue Inventory
CACI: Chemotherapy-associated cognitive impairment
CSF: Cerebrospinal fluid
CV: Coefficient of variance
ELISA: Enzyme-linked immunosorbent assay
ESBC: Early-stage breast cancer
FACT-Cog: Functional Assessment of Cancer Therapy-Cognitive Function
LTP: Long-term potentiation
MMP: Matrix metalloproteinases
SNP: Single nucleotide polymorphism
